# Scanning Electrochemical Cell Microscopy Investigation of Single ZIF‐Derived Nanocomposite Particles as Electrocatalysts for Oxygen Evolution in Alkaline Media

**DOI:** 10.1002/anie.201908021

**Published:** 2019-08-21

**Authors:** Tsvetan Tarnev, Harshitha Barike Aiyappa, Alexander Botz, Thomas Erichsen, Andrzej Ernst, Corina Andronescu, Wolfgang Schuhmann

**Affiliations:** ^1^ Analytical Chemistry—Center for Electrochemical Sciences (CES) Faculty for Chemistry and Biochemistry Ruhr University Bochum 44780 Bochum Germany; ^2^ Chemical Technology III Faculty of Chemistry and CENIDE Center for Nanointegration University Duisburg Essen Carl-Benz-Str. 199 47057 Duisburg Germany

**Keywords:** electrocatalysis, metal–organic frameworks, nanoparticles, oxygen evolution reaction, scanning electrochemical cell microscopy

## Abstract

“Single entity” measurements are central for an improved understanding of the function of nanoparticle‐based electrocatalysts without interference arising from mass transfer limitations and local changes of educt concentration or the pH value. We report a scanning electrochemical cell microscopy (SECCM) investigation of zeolitic imidazolate framework (ZIF‐67)‐derived Co−N‐doped C composite particles with respect to the oxygen evolution reaction (OER). Surmounting the surface wetting issues as well as the potential drift through the use of a non‐interfering Os complex as free‐diffusing internal redox potential standard, SECCM could be successfully applied in alkaline media. SECCM mapping reveals activity differences relative to the number of particles in the wetted area of the droplet landing zone. The turnover frequency (TOF) is 0.25 to 1.5 s^−1^ at potentials between 1.7 and 1.8 V vs. RHE, respectively, based on the number of Co atoms in each particle. Consistent values at locations with varying number of particles demonstrates OER performance devoid of macroscopic film effects.

## Graphical abstract

**The oxygen evolution reaction** (OER) activity of single and multiple ZIF‐derived Co/N−C nanocomposite particles was examined by high‐throughput scanning electrochemical cell microscopy. Through the use of an Os complex as an internal reference standard, the intrinsic activity of the catalyst with respect to the OER activity as well as the turnover frequency could be determined.

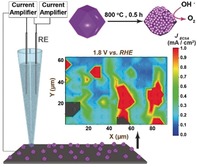

Recent advancement in earth‐abundant, non‐noble metal based electrocatalysts has stimulated visions for their utilization in sustainable energy infrastructure.^1^ The rational development of effective electrocatalysts requires a fundamental understanding of the intrinsic structure–reactivity relationship with regard to individual catalyst particles and their averaged ensemble properties.^2^ Accordingly, significant efforts have been made to analyze individual electrocatalytic profiles using nanoprobe techniques, for the extraction of current responses at small physical dimensions.^3^ The droplet cell‐based scanning electrochemical cell microscopy (SECCM) technique is one of the powerful local electrochemical tools extensively explored by Unwin and co‐workers to visualize heterogeneous electron transfer processes on single‐crystal and polycrystalline surfaces.^4^, ^5^ SECCM offers high‐resolution insight into the structure–activity relationship at the nanoscale of a heterogeneous electrochemical interface.^6^, ^7^ However, due to the necessary wetting by the positioned nanodroplet cell and the inherent difficulties of surface wetting using highly alkaline electrolytes, SECCM has been rarely applied for the investigation of electrocatalytic reactions of non‐noble metal catalysts, which requires the use of alkaline electrolytes owing to their limited stability in acidic media.^8^ We recently determined the OER activity of a single Co−N/C nanocomposite particle on top of a nanoelectrode and found a TOF of about 5 s^−1^ per Co atom, under non‐limiting mass transport conditions.^9^ Here, we report a complementary, faster, sequential, and more pragmatic approach to derive the electrochemical response of one to small numbers of ZIF‐67‐derived composite nanoparticles using SECCM.

The composite nanoparticles are inspired by carbon‐supported non‐noble metal electrocatalysts.^10^ The well‐defined molecular structure and morphology of the ZIF‐67 nanocrystals allow precise quantification of the number of Co atoms in the N‐doped carbon composite (Co−N/C).^11^ The poor stability of the commonly used miniaturized Ag‐based “quasi‐reference/counter electrode (QRCE)” systems, here chloridized silver wires, at higher current densities and at alkaline pH values leads to a considerable drift in the working electrode potential making the nanoscale voltammetric measurements unreliable.^12^ Here, we introduce a specifically designed non‐interfering, reversible redox compound as an internal potential standard to account for the potential drift during SECCM measurements at alkaline conditions. Moreover, the voltammetric response of the pH‐independent redox conversion of an Os complex (Figure 1 a) was also used to derive the relative wetted electroactive surface area at each landing site of the SECCM tip.

**Figure 1:**
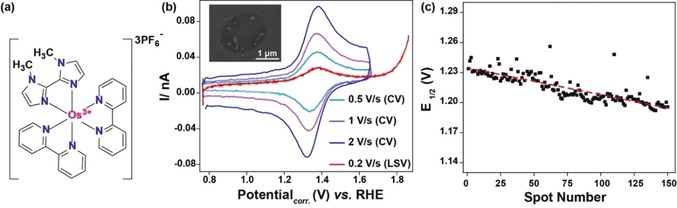
(a) The osmium(II) complex used as the internal free‐diffusing and pH‐independent redox species; (b) typical voltammograms recorded at each location after landing of the SECCM tip and corresponding SEM image of a bare measurement area on the GC surface (inset); (c) potential drift during a SECCM scan with about 150 sequential landing events corresponding to 150 individual measurement areas.

Initially, ZIF‐67 nanocrystals with a size ranging from 165 to 235 nm were deposited on a glassy carbon (GC) plate (Scheme 1 a). The GC plate was pulled out of the ZIF‐67 solution stepwise to form a gradient in surface coverage of ZIF‐67 nanocrystals (for details see Section 2, Supporting Information), and the laterally heterogeneous surface coverage of the ZIF‐67 nanocrystals was verified by means of scanning electron microscopy (SEM). Structure and phase purity of the ZIF‐67 nanocrystals were confirmed by powder X‐ray diffractometry and comparison with the simulated ZIF‐67 crystal structure^13^ (Figure S1, Supporting Information). The formation regions with varying ZIF‐67 nanocrystal densities (from single to multiple units in the landing area of the formed nanodroplet) is critical to derive the response of individual ZIF‐67‐derived nanocomposite particles as well as of few particle ensembles in a single experiment. The GC plate modified with ZIF‐67 particles was heat‐treated under an inert reducing atmosphere (H_2_/Ar) to pyrolytically transform ZIF‐67 nanocrystals into Co−N/C nanocomposites (Scheme 1 b). Local voltammetric measurements were performed by means of hopping‐mode SECCM with a predefined movement in *x*‐ and *y*‐direction of 7 μm between measurement areas. The approach was performed with the SECCM tip being vibrated normal to the sample surface (Scheme 1 c). During the approach, the currents between the two barrels of the capillary and between one of the barrels and the sample surface were simultaneously monitored. In particular, the AC component of the currents induced by the vertical vibration was used as the feedback signal. A sudden increase in the AC signal was indicative of contact between the meniscus at the end of the nanopipette and the sample surface as previously described^14^ (for details see Section 4, Supporting Information). Initial SECCM experiments using chloridized Ag wires as the QRCE displayed an irregular potential drift likely due the formation of Ag hydroxide during polarization in alkaline solution. We employed Pt wires instead and monitored and corrected for possible potential drifts by adding a soluble Os complex (Figure 1 a) to the electrolyte within the capillaries as an internal reference system. With a formal potential of 1.35 V vs. RHE for the Os^3+/2+^ redox conversion, which is ≈350 mV more cathodic than the potential at which measurable OER activity can be observed (Figure 1 b), no interference with electrocatalysis is supposed. A double‐barrel theta nanopipette pulled to a narrow elliptical tip (diameter of for example, 1.63 μm or 0.78 μm) was filled with a solution containing 0.1 mm Os^2+/3+^ complex in 50 mm KOH. These concentrations were optimized to minimize precipitation of KOH on the capillary landing positions, following the electrolyte exposure over prolonged experimental time scale (Figure S2). A Pt wire (0.3 mm diameter) was inserted into each barrel and a fixed bias potential of 20 mV was applied between the two electrodes. Upon each landing of the SECCM tip on the sample surface, three cyclic voltammograms (CV) at different scan rates were recorded in a potential range from 0.8 to 1.65 V vs. RHE. Afterwards, a linear sweep voltammogram (LSV) was recorded with a scan rate of 200 mV s^−1^ using an extended potential window to derive the OER response from each of the locations as shown in Figure 1 b. In a single array scan with multiple tip landings, electrochemical response profiles from ≈75 % of the locations could be obtained. All voltammograms showed the redox peaks corresponding to the reversible electrochemistry for the Os^3+/2+^ couple (Figure S3, Supporting Information).

**Scheme 1:**
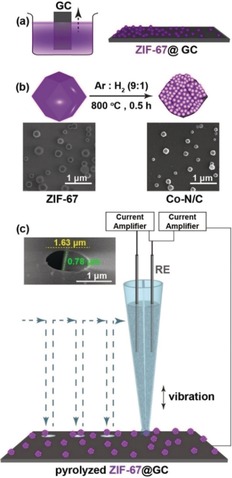
Schematic illustration of (a) ZIF‐67@GC synthesis; (b) pyrolytic transformation of ZIF‐67 nanocrystals into Co−N/C nanocomposite particles along with the corresponding SEM images; (c) typical SECCM setup and SEM image of the double‐barrel nanocapillary used as the SECCM tip (inset).

Potential drifts of up to 40 mV were measured in a single experiment (Figure 1 c). The potential‐corrected LSV curves were further corrected with respect to the capacitive double‐layer charging current (details in Section 4, Supporting Information). For a conclusive demonstration of the sequential activity mapping, SEM images of the electrochemically evaluated area were complementarily used as a “roadmap” to correlate the number of ZIF‐derived nanocomposite particles at each spot with the corresponding electrochemical response (Figure 2 a). Figure 2 b shows the increase in the number of Co atoms with the number of pyrolyzed ZIF‐67 units. The number of Co atoms (mol_Co_) within the nanocomposite particles was derived using the simulated ZIF‐67 crystallographic information considering the volume shrinkage during pyrolysis (details in Section 5, Supporting Information).

**Figure 2:**
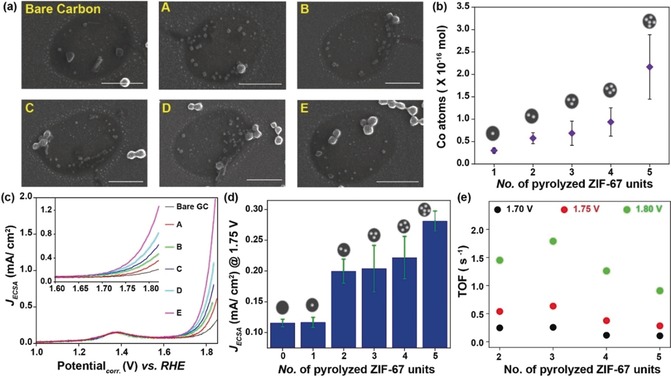
(a) Representative SEM images from locations enclosing different numbers of composite nanoparticles (scale bar=1 μm); (b) plot indicating the increase in the number of Co atoms along with the number of composite particles; (c) typical LSV recorded at the corresponding locations from (a), with an enlarged view of the OER response (inset); (d) histogram indicating the trend in OER current in accordance with the number of composite particles at 1.75 V vs. RHE; (e) evaluation of TOF at the corresponding locations at different potentials.

The geometric surface area wetted by the droplet formed upon contact of the SECCM capillary with the surface is generally assessed by measuring the size of the meniscus footprint produced during the scan, for example, by means of SEM.^4^, ^7^ However, in alkaline solution the geometric area is highly influenced by the wetting properties of the sample at the applied electrochemical potential.^15^ Determination of the exact areal contribution of individual pyrolyzed ZIF‐67 units was intricate owing to their small dimensions and varying interparticle distances. Therefore, the voltammograms were normalized with respect to their electrochemically active surface area, which was derived from the Os^3+/2+^ oxidation peak of the LSV using the Randles–Sevcik equation^16^ (details in Section 4, Supporting Information).

The voltammograms together with the corresponding SEM images showed negligible OER activity of the bare GC surface in the absence of nanocomposite particles (Figure 2 c). LSVs showed substantially higher anodic OER currents each time the nanoprobe landed on an area in which a nanocomposite particle or particle ensemble was present. A comparison of the catalytic OER activity at the measurement locations shown in Figure 2 a indicated an increase in the OER activity with the number of nanocomposite units (Figure 2 d). The locations enclosing two and three Co−N/C particles displayed a similar electrochemical activity (Figure 2 d), which may be due to different particle sizes^17^ (Figure 2 b). The intrinsic electrocatalytic activity of pyrolyzed ZIF‐67 composite particles was further evaluated in terms of the turnover frequency (TOF). Locations with a single nanoparticle exhibited marginal OER activity as compared with the bare carbon surface (Figure 2 d and Figure S9, Supporting Information) and were therefore excluded from TOF evaluation. The contribution of the carbon surface to the total measured current on each spot was subtracted to investigate the catalytic activity of only the Co−N/C nanocomposite particles (calculation details in Section 6, Supporting Information).

The TOF derived at three different potentials (1.70, 1.75, and 1.80 V vs. RHE) reflected the expected increase in the OER activity with increasing anodic potentials, with values of about 0.25 to 1.5 s^−1^ per Co atom (Figure 2 e). The OER activity is influenced by interfacial pH, local O_2_ supersaturation, and blocking of the catalytic surface by O_2_ gas bubbles.^18^ The decrease in TOF with an increase in the number of nanocomposite particles reflects the increasing variations in the chemical environment within the droplet. A spatially resolved electrochemical movie was compiled using an array scan comprising about 150 measurement areas and hence ≈150 LSVs in the potential range from 1 to 1.8 V vs. RHE. The electrochemical reactivity maps derived at potentials in the OER region are in good agreement with the particle coverage on the GC surface, as confirmed using the complementary SEM images (Figure 3).

**Figure 3:**
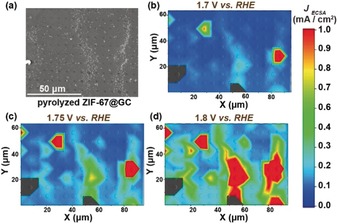
(a) SEM image of pyrolyzed ZIF‐67@GC used for the SECCM investigation. Representative SECCM movie frames at different potentials (b) 1.7 V, (c) 1.75 V, and (d) 1.8 V vs. RHE, respectively. The measured spots are marked with circles, the colors in the areas between them are interpolated by the graphics software.

In conclusion, we report a versatile, effective, high‐throughput SECCM measurement to derive the electrochemical reactivity of a single to several individual Co−N/C composite nanoparticles in a single experiment at alkaline conditions. Using a free‐diffusing redox compound as an internal potential standard allowed to compensate not only for potential drifts but also to derive the wetted surface area for each individual droplet formed by the landing of the SECCM tip as the basis to derive the OER activity and the TOF of individual Co−N/C nanocomposite particles.

## Conflict of interest

The authors declare no conflict of interest.

## Supporting information

As a service to our authors and readers, this journal provides supporting information supplied by the authors. Such materials are peer reviewed and may be re‐organized for online delivery, but are not copy‐edited or typeset. Technical support issues arising from supporting information (other than missing files) should be addressed to the authors.

Supplementary
