# Supplementary material for: Scanning Electrochemical Cell Microscopy Investigation of Single ZIF‐Derived Nanocomposite Particles as Electrocatalysts for Oxygen Evolution in Alkaline Media
Source: Angew Chem Int Ed Engl. 2019 Aug 21;58(40):14265–9. doi: 10.1002/anie.201908021 (PMC6790716; doi:10.1002/anie.201908021)
Supplement: Supplementary file 1 — Supplementary [file ANIE-58-14265-s001.pdf]

Angewandte Chemie
Eine Zeitschrift der Gesellschaft Deutscher Chemiker

Supporting Information

# Scanning Electrochemical Cell Microscopy Investigation of Single ZIF-Derived Nanocomposite Particles as Electrocatalysts for Oxygen Evolution in Alkaline Media

*Tsvetan Tarnev*[+]*, Harshitha Barike Aiyappa*[+]*, Alexander Botz, Thomas Erichsen, Andrzej Ernst, Corina Andronescu,* and Wolfgang Schuhmann**

anie_201908021_sm_miscellaneous_information.pdf

**Table of Contents**


| Section No. | Title | Page No. |
|---|---|---|
| 1 | Experimental | 1 |
| 2 | Sample preparation and structural characterization | 3 |
| 3 | Quantification of the potential drift using the Osmium complex | 4 |
| 4 | SECCM experiments | 6 |
| 5 | Quantification of Co atom within each ZIF-derived nanocomposite | 11 |
| 6 | TOF evaluation | 11 |
| 7 | References | 12 |


## Section 1. Experimental

### 1.1 Chemicals and Materials

Methanol, cobalt(II)nitrate hexahydrate and 2-methylimidazole were purchased from Sigma-Aldrich; KOH was obtained from Roth. Ethylene glycol and ammonium hexafluorophosphate were purchased from ACROS Organics, Fisher Scientific, and Alfa Aesar. All chemicals were used without further purification. Double-barrel quartz capillaries (0.9 mm inner diameter, 1.2 mm outer diameter, 10 cm length) with filaments (WAR-QTF-120-90) were purchased from Friedrich & Dimmock.

### 1.2. Instrumentation

**Scanning electron microscopy** (SEM) micrographs were recorded using a Quanta 3D ESEM (FEI) microscope at 20 kV in the high vacuum mode.

**Scanning electrochemical cell microscopy (SECCM) set-up**

The SECCM system consists of positioning system equipped with 3 stepper motors (Owis) with a LStep PCIe (Lang) controller for coarse positioning and a x,y,z-piezocube (P-611.3S nanocube, Physik Instrumente) for fine positioning using an analogue amplifier (E-664, Physik Instrumente). The sample is fixed to the nanocube. The probe is vibrated with an additional piezo rod (PSt 150/5/60 VS10, Piezomechanik) with an amplifier also from Piezomechanik (LE150/100/EBW). The AC perturbation signal for piezo vibration is generated by the internal oscillator of a lock-in amplifier (7280 DSP, Signal Recovery). Current measurements are performed with two current amplifiers. One of them (VA-10, NPI) is measuring the current between the quasi-reference counter electrodes (QRCEs) in the barrels of the

capillary, the other (ELC-03XS, NPI) between one of the QRCEs and the sample. Both amplifiers are using the same Pt wire as reference. The measured current between the barrels is forwarded to the lock-in amplifier to extract the AC component. The current amplifiers and the amplifier for the piezo nanocube are controlled by a FPGA card (PCIe-7852R, National Instruments), connected to a personal computer using a break-out box (SCB-68A, National Instruments) with a SHC68-68-RMIO (National Instruments) cable. The FPGA card is also used to collect the measured data from the current amplifiers, the lock-in amplifier and the nanocube amplifier. The software running on the FPGA card and on the PC is written in LabVIEW (National Instruments) and is a modified version of a software provided by the University of Warwick (WEC-SPM). The whole set-up is built on a vibration damping table (RS 2000, Newport) set on four S-2000 stabilizers (Newport) to decouple it from oscillations of the building. The set-up is placed in a Faraday cage equipped with thermal isolation panels (Vaku-Isotherm) to prevent any temperature drift during measurements. Additional components are a video microscope camera (DMK 21AU04, The Imaging Source) to facilitate the initial positioning of the SECCM tip and a cold light source (KL1500 LCD, Schott).

### 1.3 Preparation of the double barrel nanopipette

A quartz theta capillary was surface cleaned using acetone and pulled by means of a laser puller (P-2000, Sutter Instruments, USA) using a two line program: Line 1: Heat 800, Filament 4, Velocity 40, Delay 130, Pull 0; Line 2: Heat 650, Filament 3, Velocity 30, Delay 130, Pull 90. To render the outer walls of the pulled capillary hydrophobic, the tapered end was dipped for 30 s in dichlorodimethylsilane (Acros Organics), while applying 6.5 bar of Ar pressure from the back-end of the capillary. The capillary was dried in air for 1 min while keeping the Ar pressure. Two Pt wires (0.3 mm diameter) were used as QRCEs. In order to remove possible surface contaminants, the wires were flame heated and dipped for 1-2 s into aqua regia (***Caution!*** *Aqua regia solution is highly corrosive and a powerful oxidizing agent. This solution must be handled with extreme care*) followed by a wash with water. The capillary was then filled with a solution containing 0.1 mM [(Os (2, 2’-bipyridine)$_2$(N, N’-dimethyl-2, 2´-biimidazole)] $(PF_6)_3$ in 50 mM KOH using a MicroFil needle with 0.1 mm inner diameter (MF34G-5, World Precision Instruments). The back end of the capillary was flame-heated for 1-2 s and silanized to render the back-end of the capillary hydrophobic to avoid any electrolyte short-circuiting. After inserting the QRCEs into the two barrels, a CV was recorded between the two QRCEs in the potential range from -50 to 50 mV with a scan rate 50 mV/s to check the connection between them (CV of a resistor, straight line, is expected).

## Section 2. Sample preparation and structural characterization

### 2.1. ZIF-67 synthesis

10 mL of 0.05 M $Co(NO_3)_2$ in methanol were mixed with 10 mL of 0.4 M 2-methylimidazole in methanol. After 10 min of reaction, the mixture was centrifuged at 4000 rcf for 10 min. The clear liquid phase was decanted and the precipitate was dispersed in 10 mL methanol. This process was repeated four times. Additional 30 mL methanol were added to the mixture followed by sonication for 5 min. Half of the resulting solution was diluted with another 20 mL methanol to obtain the final ZIF-67 dispersion, which was thereafter used for modification of the GC plates.

### 2.2. Structural evaluation of the as-synthesized ZIF-67 nanocrystals

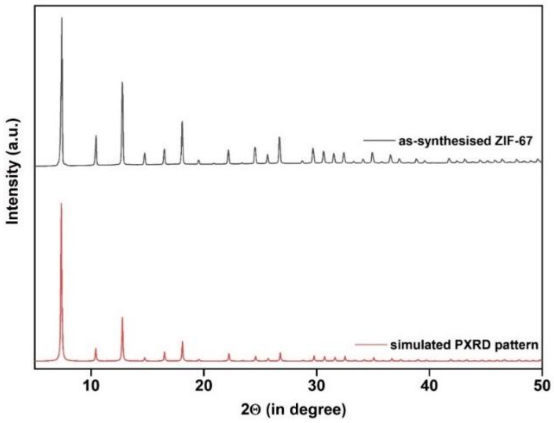


**Fig. S1** PXRD patterns of the ZIF-67 nanocrystals with that of the simulated crystal structure.

### 2.3. Preparation of bare glassy carbon (GC) plate

A GC plate was polished with diamond suspension with particle sizes of 3, 1, 0.5 and 0.1 µm (1.5 min polishing with each particle size). Afterwards, the GC plate was sonicated for 10 min in water : ethanol mixture (1:1) and then dried under a steady Ar flow.

### 2.4. Dispersion of ZIF-67 nanocrystals on the GC substrate and their pyrolytic transformation

The cleaned GC plate (20 mm × 10 mm) was submerged 14 mm into 3 mL of the ZIF-67 precursor solution. It was retracted stepwise (2 mm steps) with one minute waiting time at each step to achieve an increasing coverage with ZIF-67 crystals. The GC plate was heated in a three-zone furnace to pyrolyze the ZIF-67 nanoparticles under formation of Co/N-C nanocomposite particles. The heating sequence was: 30 min flushing with Ar at 30°C; heating up to 800°C with 10 °C/min in an atmosphere of 90 % Ar and 10 % $H_2$; 30 min pyrolysis at 800°C in an atmosphere of 90 % Ar and 10 % $H_2$; cooling down for 25 h in 100 % Ar. The GC plate was contacted with self-adhesive Al tape with conductive glue (3M).

## Section 3. Quantification of the potential drift using an Osmium complex

### 3.1 Implementation of Pt wires as QRCE and the conversion of its potential to RHE

Chloridized Ag wires as quasi-reference/counter electrodes have been shown to produce stable and reliable reference potentials in acidic media [1]. However, in the case of alkaline electrolytes, the potentials drift gradually from the initial value, presumably due to formation of $Ag_xO$ species on the surface of the wires.[2] In the present study, initial SECCM experiments using chloridized Ag wires as the QRCE displayed an irregular potential drift. Hence, more stable Pt wires were used as QRCE. The potentials were measured versus the Pt wire in one of the barrels of the capillary but converted to reversible hydrogen electrode (RHE) according to the following equation: $E_{RHE} = E_{Pt} + OCP_{Pt} + E^{o}_{Ag/AgCl} + 0.059 * pH$, where $OCP_{Pt}$ is the open circuit potential of the Pt wire measured in 0.1 M KOH versus *Ag/AgCl/3 M KCl* before the scan and $E^{o}_{Ag/AgCl}$ = 0.210 V *vs.* NHE in 3 M KCl solution at 25°C.

It is important to note that during the evaluation of the OER activity, LSVs were recorded at each grid points. Therefore, according to the typical two-electrode format, the reference-counter Pt wire would be under reducing potentials. As the Pt wire is stable under reducing potential, the OER activity assessment from the scan points is perceived to be free from interference of any possible Pt-decomposition products.

### 3.2. Optimization of SECCM parameters

SECCM experiments performed using higher concentration of the electrolyte (> 0.05 M KOH) and lower scan rates (< 200 mV/s) were observed to induce precipitation of KOH on the measurement areas, thereby masking the visibility of the meniscus footprints in the SEM. To preserve the meniscus footprint for a post-SECCM SEM analysis, the measurements were performed using 0.05 M KOH at a scan rate of 200 mV/s.

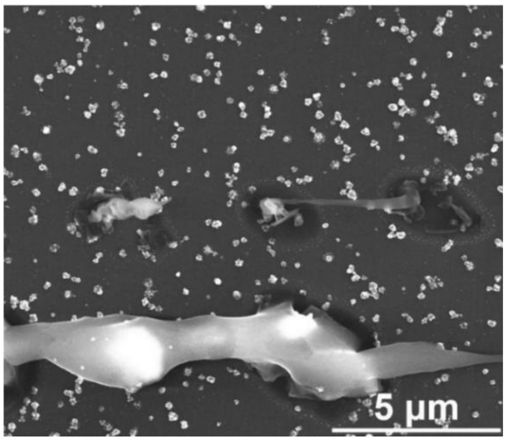


**Fig. S2** SEM image of an area containing several landing spots of the SECCM tip. At each measuring area a CV with a scan rate of 50 mV/s was performed leading to drying of the electrolyte at the landing spots. QRCE: chloridized silver wire; electrolyte: 0.1 M KOH.

**3.3 Synthesis of [(Os (2, 2'-bipyridine)$_2$(N, N'-dimethyl-2,2´-biimidazole)]**

The Os-complex and the ligand used in the following step was synthesized using a previously reported protocol. Os(2,2'-bipyridine)$_2$Cl$_2$ [3] (314 mg, 0.548 mmol) and the ligand N,N'-dimethyl-2,2´-biimidazole [4](107 mg, 0.66 mmol) were dissolved in deaerated ethylene glycol under argon atmosphere and heated to 150 °C in a closed vessel. The slurry was stirred at this temperature overnight and then cooled down to room temperature and quenched with 50 mL of water containing 0.8 g $NH_4PF_6$ to precipitate the product as the $PF_6$ salt at 0 °C. The dark product was separated by centrifugation and washed with water. Finally, the combined fractions were dried in vacuum to yield a brown powder. Yield: 478 mg (79 %). *E* = 0.393 V *vs. Ag/AgCl/3 M KCl*; the Os complex was drop cast onto a GC electrode after dissolution in acetone. Electrolyte: 1 M KCl in water. UV-vis (in DMSO): λ/nm 292 (max) with a broad shoulder at ≈ 360.

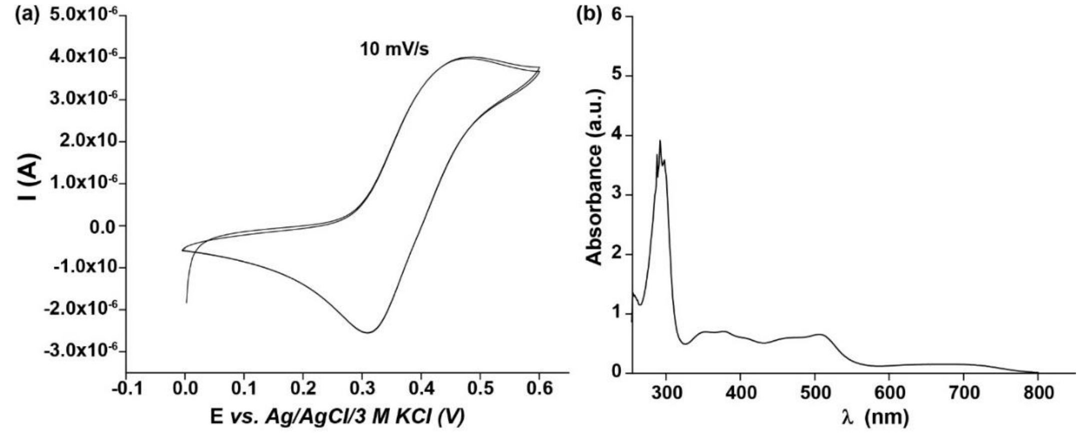


**Fig. S3** (a) CV and (b) UV-Vis absorption spectrum of the synthesized Os (II) complex.

**3.4 Diffusion coefficient of the Os (II) complex**

A GC disc electrode (2 mm radius) was polished using diamond suspension with particle sizes of 3, 1 and 0.5 µm (1.5 min polishing with each particle size) and afterwards sonicated for 10 min in $H_2O$:ethanol = 1:1. The clean GC was submerged in the solution containing 0.1 mM [(Os(2, 2'-bipyridine)$_2$(N,N'-dimethyl-2,2´-biimidazole)](PF$_6$)$_3$ in 50 mM KOH (same as used for the SECCM measurements) with a Pt-mesh counter electrode and a Ag/AgCl/3 M KCl reference electrode. The potential was stepped from 0 to 0.5 V *vs.* Ag/AgCl/3 M KCl and the current response was measured. From the slope (derived using linear regression) of the plot of the current, I, versus $t^{-1/2}$ (**Fig. S4** ) between 10 and 30 s after the potential step, the diffusion coefficient, D, of the Os complex was calculated using the Cottrell equation [5] to be D = $6.14*10^{-5}$ cm$^2$/s.

$$Slope = \frac{n * F * A * C\sqrt{D}}{\sqrt{\pi}}$$

With n = number transferred electrons, F = Faraday constant, A = area of the electrode, C = concentration of the electroactive species, D = diffusion coefficient.

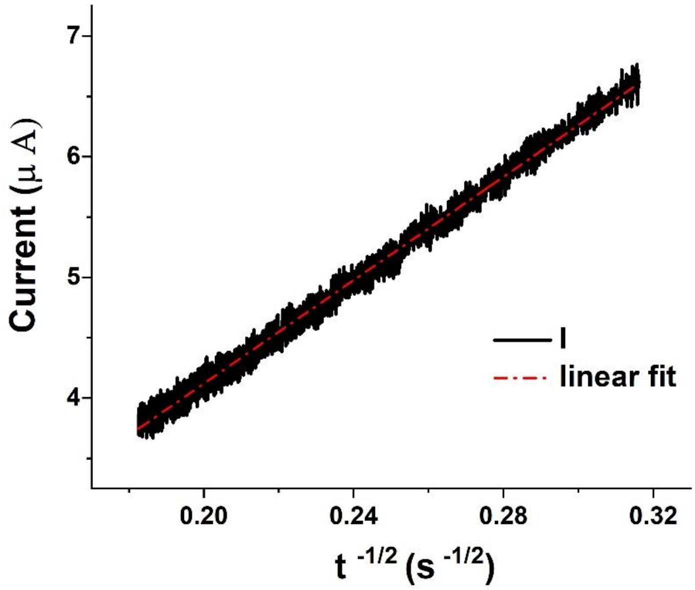


**Fig. S4** Plot of current *vs.* $t^{-1/2}$ between 10 s and 30 s after the potential step from 0 to 0.5 V vs Ag/AgCl /3 M KCl was applied to the GC disk electrode in the presence of the Os-complex (black line). The linear regression line is shown in red.

## Section 4. SECCM experiments

### 4.1. Positioning of the nanopipette on the substrate

The filled capillary with the QRCEs was positioned with the help of an optical microscope camera at a distance of approximately 10 µm above the sample surface. The piezo nanocube was programmed to approach the surface at a rate of 0.1 µm/s, while a voltage of 0.02 V is applied between the barrels of the capillary and a potential of 0 V at the GC surface (evidently, at that time the GC surface is not connected with the capillary and therefore it is not part of a closed electric circuit). During the approach the capillary oscillated in the direction normal to the GC surface with an amplitude of 40 nm at a frequency of 70 Hz. The AC component of the current measured between the barrels was extracted using a lock-in amplifier and it was used as the feedback criterion to stop the approach if the magnitude of the AC current signal exceeded 1 mV at the analogue output of the lock-in amplifier. Typically, 2-3 approaches to the surface until landing were performed and corresponding CVs were recorded to test the stability of the signals before the commencement of the SECCM scan in hopping mode. The scans were performed using parameters similar to that during the approach with an exception that a potential of -0.7 V was applied between the GC surface and one of the Pt-wires (used as QRCE) during each approach and retraction. The magnitude of the AC component of the signal between the barrels was used as a feedback criterion, with a threshold value of 1 mV (a typical approach curve is represented in Fig. S5). Upon stopping the approach on each point of the scan, three CVs (in the potential range from -0.7 to 0.2 V, 2 cycles each with 0.5, 1.0 and 2.0 V/s, respectively) and one LSV (in the potential range from -0.7 to 0.4 V, 0.2 V/s) were recorded. The capillary was then retracted up to a distance of 4 µm and translated

laterally to the next position of the scan grid (overall dimensions 98 µm x 98 µm with an increment of 7 µm in both x- and y-directions), where the next approach was started using the same procedure.

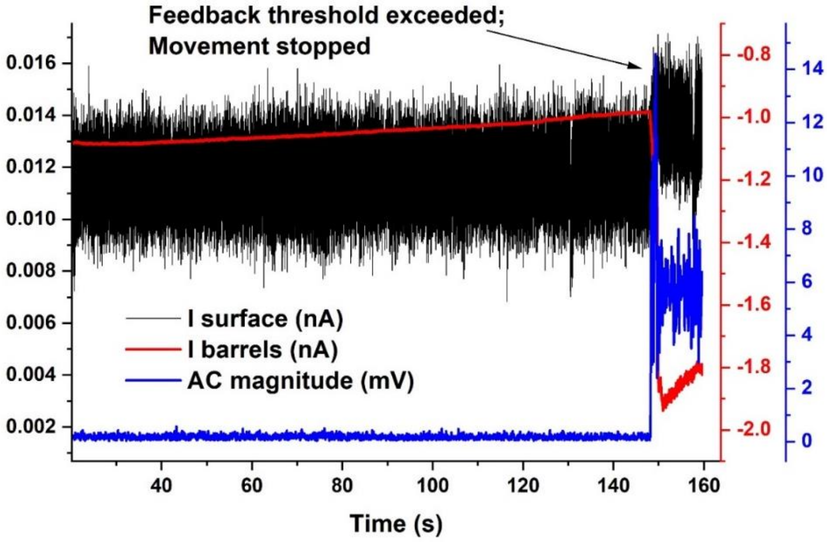


**Fig. S5** SECCM approach curve recorded before a scan

#### 4.2. Data treatment

The measurements from the first few grid points were found to be inconsistent. It appears that several landings of the capillary on the surface are needed before a steady droplet cell is constituted. The first five lines of the SECCM scan were therefore not considered for evaluation. However, the subsequent SECCM measurements produced consistent responses, as reflected by the CVs and the LSVs recorded at each grid point, except for two points, in which the capillary failed to land on the substrate due to false feedbacks. The two points are marked black in SECCM movie frames.

All voltammograms were smoothed with a moving averaging window of 5 points. Typically, the potentials in the middle between the peak potentials of all CVs were found ($E_{1/2}$) and thereafter used to correct the respective CVs and LSVs for the potential drift by referring to the formal potential value of the used Os complex (0.393 V *vs.* Ag/AgCl/3 M KCl, which corresponds to 1.35 V *vs.* RHE according to the equation $E_{RHE} = E_{Ag/AgCl} + E^0_{Ag/AgCl} + 0.059 * pH$ with $E^0_{Ag/AgCl} = 0.210$ V *vs.* NHE for 3 M KCl solution at 25°C). Each CV was corrected individually, whereas for the correction of the LSVs the calculated $E_{1/2}$ values of all three CVs from the same measuring area were averaged and the difference of the averaged value and the formal potential of the complex was subtracted from the LSV potentials.

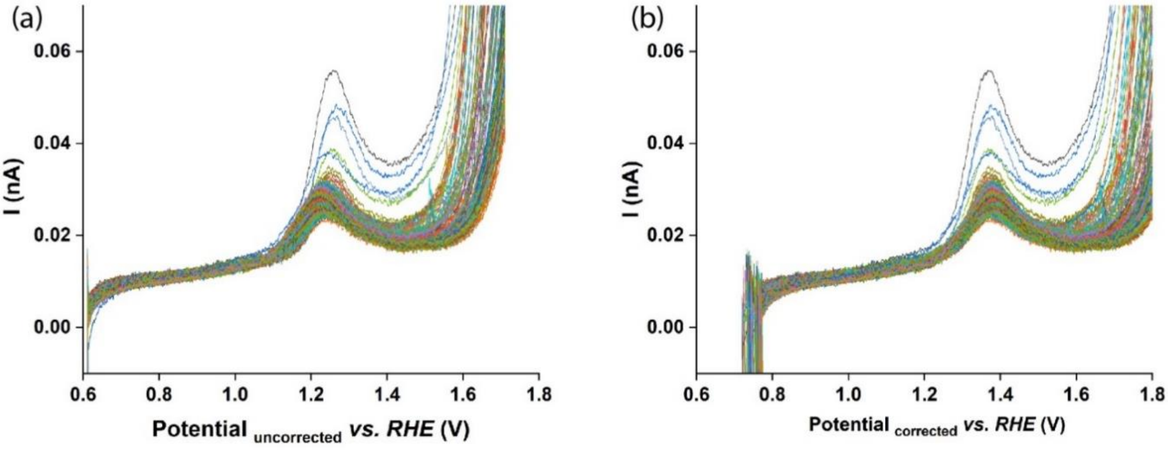


**Fig. S6** LSVs from different measurement areas of a SECCM scan (a) before and (b) after potential drift correction (scan rate: 0.2 V/s).

Capacitive current correction: Regression lines were calculated between 0.8 and 0.95 V *vs.* RHE for the anodic and cathodic sweep of each CV. For each spot of the SECCM scan the current values at 0.85 V *vs.* RHE of each CV were plotted versus the scan rate and extrapolated to 0.2 V/s (scan rate of the LSV) for both the anodic and cathodic sweeps. The capacitive current of the LSV is the average of the two obtained values at 0.2 V/s and is subtracted from the current values of the LSV.

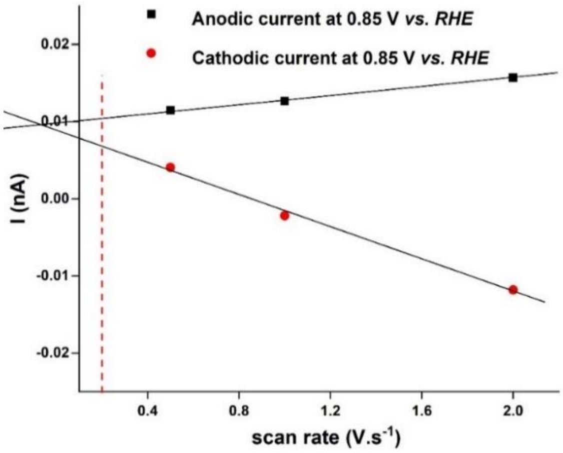


**Fig. S7** Determination of the capacitive current of the LSV at 0.2 V/s by extrapolation of the capacitive currents of the three CVs on each spot

Normalization of the LSV currents: The potential and capacitive current corrected LSVs were normalized with respect to the surface area calculated with the Randles-Sevcik equation [5]:

$$i_p = 0.4463 * n * F * A * C * \sqrt{\frac{n * F * \nu * D}{R * T}}$$

Where, $i_p$ = peak current of the LSV; n = number of transferred electrons; F = Faraday constant; A = area; C = concentration; v = scan rate; D = diffusion coefficient; R = universal gas constant; T = temperature.

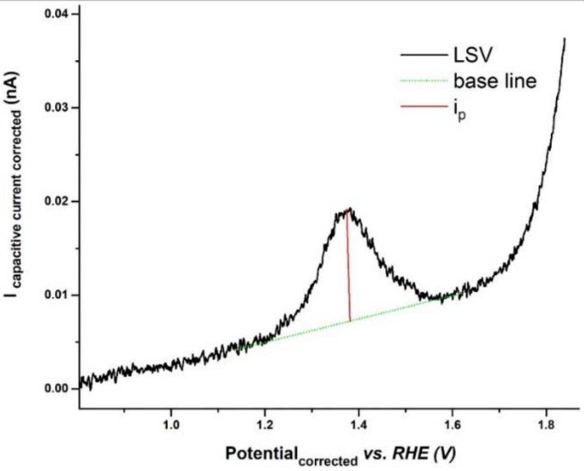


**Fig. S8** Example for determination of the peak potential of the LSVs (scan rate: 0.2 V/s).

Os-complexes are typical fast, outer-sphere redox mediators with high chemical stability, fast reversibility and pH-independent activity, which makes them prospective internal standards for potential measurements. The chosen Os-complex exhibits a formal potential of 1.35 V vs. RHE for the $Os^{3+/2+}$ redox conversion, which is ≈ 350 mV more cathodic than the potential at which measurable OER activity can be observed. The SECCM scans on bare glassy carbon surface show negligible OER activity, while a distinct increase in cell currents are observed in locations enclosing ZIF-derived composite units. As the droplet cell contains internal redox mediator in both the cases, it is also demonstrating the poor activity of the Os-complex towards catalyzing OER. Although it is intricate to determine the influence of the Os-complex on the ZIF-derived composite's electrocatalytic activity, the chemical inertness of Os-complexes prevents interaction with ZIF-derived composite and thereafter influence on its electrocatalytic activity.

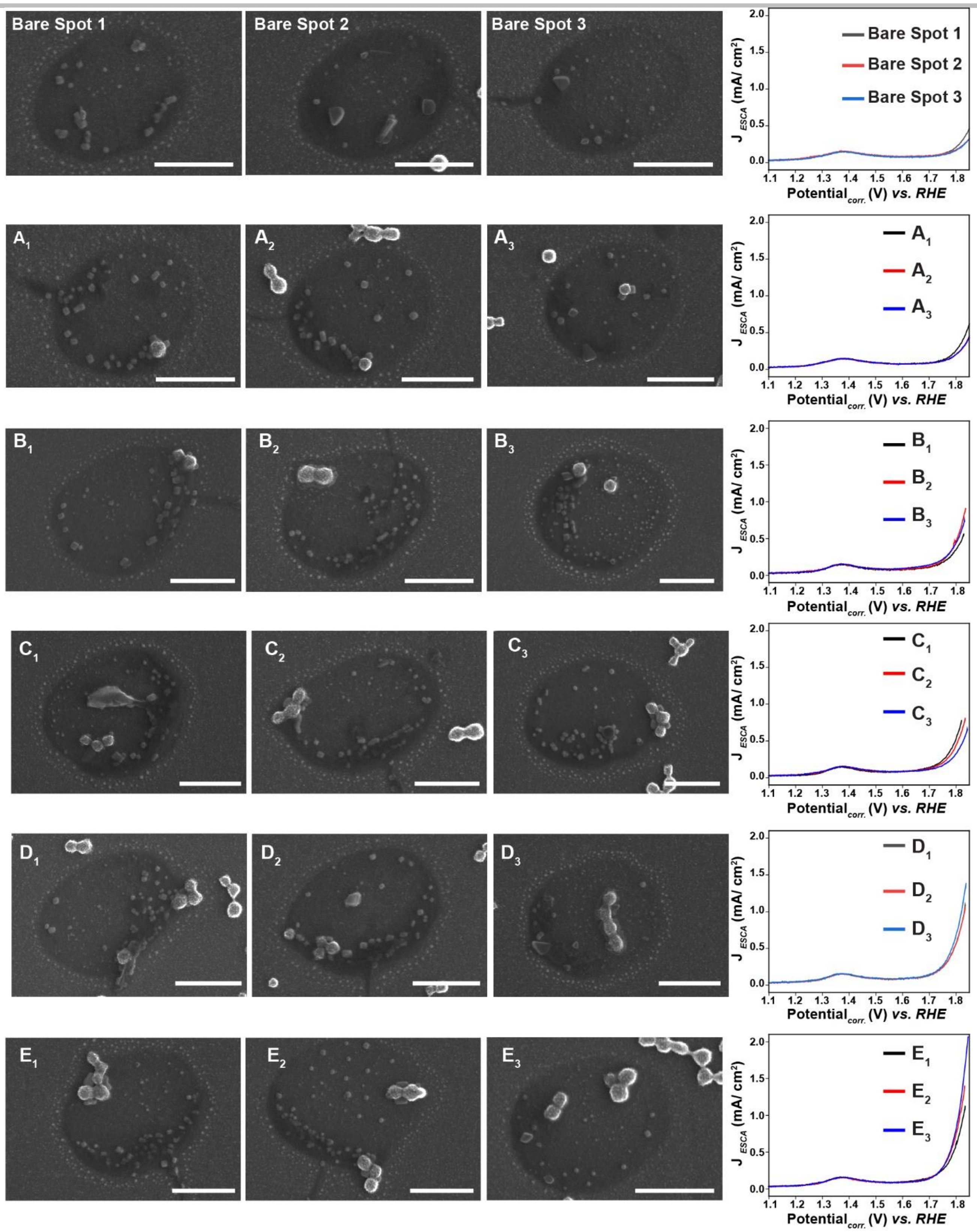


**Fig. S9** SEM images from SECCM measuring areas containing varying numbers of ZIF-67-derived Co/N-C nanocomposite particles (varying from 0 to 5 units) and the corresponding LSVs recorded at a scan rate of 0.2 V/s.

## Section 5. Quantification of the number of Co atoms within each ZIF-derived nanocomposite

### 5.1 Calculation of the shrinkage of ZIF-67 nanocrystal during pyrolysis

The percentage shrinkage of the particles during pyrolysis was calculated by measuring the edges of some particles before and after pyrolysis by means of SEM (Fig. S14). The average shrinkage was determined to be 31 ± 3 %.

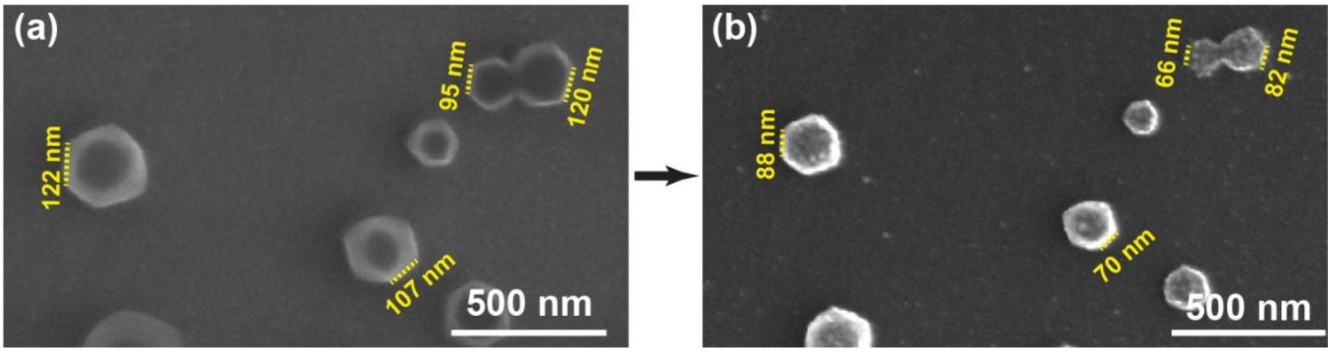


**Fig.S10** Representative SEM images indicating the shrinkage in the volume of the ZIF-67 nanocrystals (a) before and (b) after pyrolysis.

### 5.1. Estimation of amount of Co atoms within each ZIF-derived nanocomposite

The size of the ZIF-nanocrystal before pyrolysis was assessed by recalculating with the % shrinkage at the employed pyrolytic conditions (section 5.1). The volume of the crystal with an edge length ‘a’ was thereafter used to calculate the amount of Co atoms within each nanocrystal:

$$V_{rhombic\ dodecahedron} = \frac{16 * \sqrt{3}}{9} * a^3$$

A single unit cell of ZIF-67 (SOD) contains 12 Co atoms and has a volume 4877.5 × $10^{-24}$ $cm^3$ according to the simulated structure [6]. With this information the number of unit cells in a pyrolyzed ZIF-67 nano-composite can be calculated, from which the number of Co atoms and consequently the mol amount of Co are accessible.

## Section 6. TOF evaluation

To calculate the TOF, the contribution of the GC surface to the overall current is subtracted according to the following formula:

$$I_{corrected} = I_{total} - j_{GC} * ECSA_{spot}$$

where, $I_{corrected}$ = GC surface contribution corrected current; $I_{total}$ = total current of the spot and $j_{GC}$ = current density of bare GC (as calculated in section 4.2) at given potential; $ESCA_{spot}$ = electrochemically active surface area of the spot (as calculated in section 4.2).

The TOF is evaluated under the assumption that no Co atoms are lost during pyrolysis, according to:

$$\mathrm{TOF} = \frac{\mathrm{I}}{4 * \mathrm{mol}_{Co} * \mathrm{F}}$$

where, I = current in the LSV at a chosen potential; $\mathrm{mol}_{Co}$ = amount of Co atoms in mol; F = Faraday constant (96485 C/mol).

**Section 7. References**

[1] C. L. Bentley, D. Perry, P. R. Unwin, *Anal. Chem.* **2018**, *90*, 7700.

[2] a) D. Shin, B. Jeong, M. Choun, J. D. Ocon, J. Lee, *RSC Adv.* **2015**, *5*, 1571; b) C. A. Campos-Roldán, R. G. González-Huerta, N. Alonso-Vante, *J. Electrochem. Soc.* **2018**, *165*, J3001-J3007.

[3] P. Pinyou, A. Ruff, S. Pöller, S. Ma, R. Ludwig, W. Schuhmann, *Chem. Eur. J.* **2016**, *22*, 5319.

[4] a) S. Teanphonkrang, S. Janke, P. Chaiyen, J. Sucharitakul, W. Suginta, P. Khunkaewla, W. Schuhmann, A. Ruff, A. Schulte, *Anal. Chem.* **2018**, *90*, 5703; b) S. Teanphonkrang, A. Ernst, S. Janke, P. Chaiyen, J. Sucharitakul, W. Suginta, P. Khunkaewla, W. Schuhmann, A. Schulte, A. Ruff, *ACS Sens.* **2019**, *4*, 1270.

[5] A. J. Bard, L. R. Faulkner, *Electrochemical Methods: Fundamentals and Applications*, Wiley, **2001**.

[6] R. Banerjee, A. Phan, B. Wang, C. Knobler, H. Furukawa, M. O'Keeffe, O. M. Yaghi, *Science (New York, N.Y.)* **2008**, *319*, 939.
